# Comparison of Two Theory-Based, Fully Automated Telephone Interventions Designed to Maintain Dietary Change in Healthy Adults: Study Protocol of a Three-Arm Randomized Controlled Trial

**DOI:** 10.2196/resprot.3367

**Published:** 2014-11-10

**Authors:** Julie A Wright, Lisa M Quintiliani, Gabrielle M Turner-McGrievy, Jeffrey P Migneault, Timothy Heeren, Robert H Friedman

**Affiliations:** ^1^Department of Exercise and Health SciencesUniversity of Massachusetts BostonBoston, MAUnited States; ^2^Medical Information Systems UnitSection of General Internal MedicineBoston University School of MedicineBoston, MAUnited States; ^3^Department of Health Promotion, Education, and Behavior DiscoveryArnold School of Public HealthUniversity of South CarolinaColumbia, SCUnited States; ^4^Warren Alpert Medical SchoolBrown UniversityProvidence, RIUnited States; ^5^Department of BiostatisticsBoston University School of Public HealthBoston, MAUnited States

**Keywords:** maintenance, social cognitive theory, goals, fruit, vegetable, diet, telephone, health information systems

## Abstract

**Background:**

Health behavior change interventions have focused on obtaining short-term intervention effects; few studies have evaluated mid-term and long-term outcomes, and even fewer have evaluated interventions that are designed to maintain and enhance initial intervention effects. Moreover, behavior theory has not been developed for maintenance or applied to maintenance intervention design to the degree that it has for behavior change initiation.

**Objective:**

The objective of this paper is to describe a study that compared two theory-based interventions (social cognitive theory [SCT] vs goal systems theory [GST]) designed to maintain previously achieved improvements in fruit and vegetable (F&V) consumption.

**Methods:**

The interventions used tailored, interactive conversations delivered by a fully automated telephony system (Telephone-Linked Care [TLC]) over a 6-month period. TLC maintenance intervention based on SCT used a skills-based approach to build self-efficacy. It assessed confidence in and barriers to eating F&V, provided feedback on how to overcome barriers, plan ahead, and set goals. The TLC maintenance intervention based on GST used a cognitive-based approach. Conversations trained participants in goal management to help them integrate their newly acquired dietary behavior into their hierarchical system of goals. Content included goal facilitation, conflict, shielding, and redundancy, and reflection on personal goals and priorities. To evaluate and compare the two approaches, a sample of adults whose F&V consumption was below public health goal levels were recruited from a large urban area to participate in a fully automated telephony intervention (TLC-EAT) for 3-6 months. Participants who increase their daily intake of F&V by ≥1 serving/day will be eligible for the three-arm randomized controlled trial. A sample of 405 participants will be randomized to one of three arms: (1) an assessment-only control, (2) TLC-SCT, and (3) TLC-GST. The maintenance interventions are 6 months. All 405 participants who qualify for the trial will complete surveys administered by blinded interviewers at baseline (randomization), 6, 12, 18, and 24 months.

**Results:**

Data analysis is not yet complete, but we hypothesize that (1) TLC-GST > TLC-SCT > control at all follow-up time points for F&V consumption, and (2) intervention effects will be mediated by the theoretical constructs (eg, self-efficacy, goal pursuit, conflict, shielding, and facilitation).

**Conclusions:**

This study used a novel study design to initiate and then promote the maintenance of dietary behavior change through the use of an evidence-based fully automated telephony intervention. After the first 6 months (the acquisition phase), we will examine whether two telephony interventions built using different underlying behavioral theories were more successful than an assessment-only control group in helping participants maintain their newly acquired health behavior change.

**Trial Registration:**

Clinicaltrials.gov NCT00148525; http://clinicaltrials.gov/ct2/show/NCT00148525 (Archived by Webcite at http://www.webcitation.org/6TiRriJOs).

## Introduction

Lifestyle behaviors, including smoking cessation, prevention of overweight and obesity, physical activity, and healthful diets, are recommended for health promotion and disease prevention across a wide range of chronic conditions including cardiovascular disease, diabetes, and cancer [1]. Epidemiological studies have examined both overall eating patterns and intake of individual foods and nutrients for their effects on overall mortality and specific diseases [2-4]. While research examining relationships between diet and disease is complex due to issues of measurement, self-reporting bias, integration of foods within the total diet, confounding, among other issues, a large compilation of research supported the rationale that particular diet behaviors can affect diet-related cancer risk, including probable evidence of decreased risk with intake of foods high in dietary fiber (colorectal cancer) and fruits and vegetables (mouth, pharynx, larynx, esophagus, and stomach cancers) [3].

Surveillance of eating patterns in the United States indicates that the majority of the population does not meet recommendations for multiple dietary components, including fruits and vegetables (F&V), which are consumed at approximately half of recommended levels [5]. A comprehensive review of 45 studies [6] provides evidence that specific dietary interventions can lead to modest effects on improving diet. For interventions that targeted F&V intake, the average amount of change has ranged from about a 0.5 serving to slightly over one serving per day increase [6-13]. Given a large cohort study that found a 53% higher mortality rate among those who consume no fruits and vegetables compared with those who eat 5 servings a day as well as a dose-response relationship between increasing levels of F&V intake and overall mortality [2], it is expected that even modest increases in F&V intake would lead to beneficial mortality outcomes.

In recent years, multiple commentators have called for the need to sustain short-term health behavioral intervention effects by studying intervention effects at the end of the intervention period as well as long-term follow-up after the intervention concluded. Gaining a better understanding of behavior change maintenance was highlighted by Kumanyika et al more than 10 years ago [14] and more recently by the Health Maintenance Consortium in 2010 [15-18]. A systematic review of the maintenance of dietary change found that while 90% of the trials reported significant outcomes at the end of the intervention, 35% reported diet and/or physical activity significant outcomes at least 3 months after the intervention ended. However, of the seven diet trials that reported long-term outcomes (range 3-12 months following the conclusion of the intervention), there were promising effects on dietary outcomes, with six trials reporting significant between group differences (ie, maintenance) of at least one dietary outcome [19]. Additional dietary interventions have been published since the time of that review, which reported significant maintenance of effects ranging from 6 months to a year after the end of the diet intervention [20-23]. For example, a telephone-based intervention using the Get Healthy Information and Coaching Service in Australia demonstrated that a 6-month intervention followed by 6 months of no contact, participants maintained their increase in fruit intake and decreases in weight, waist circumference, and body mass index (BMI) [22]. In another telephone-based intervention, intervention effects for percent calories from total fat and saturated fat, fiber, and fruit were maintained after a 12-month intervention followed by 6 months of no contact [21]. Interestingly, for both telephone-delivered interventions, maintenance of effects for vegetable intake was not sustained during the periods of no intervention contact despite initial improvements immediately following the interventions.

One aspect limiting the further development of the science of behavior change maintenance is the lack of theories that have been developed to specifically address maintenance versus initiation of a health behavior change. Some experts suggest that a new theoretical approach may be needed to understand the maintenance of behavior changes and how to design interventions [24,25]. Rothman proposes that maintenance of a behavior change may involve different cognitive processes than initiation of behavior change [26]. Theories that have been used to inform behavioral interventions (eg, social cognitive theory) may fall short in that they use the same processes to initiate and to maintain a behavior change.

The present study conceptualizes maintenance as a distinct phase of the health behavior change process and proposes Goal Systems Theory (GST) [27-30] as a possible theory to inform the design of a maintenance intervention to help individuals manage their newly acquired behavior (increased consumption of F&V) following the acquisition of this behavior. This novel approach will be compared to a widely accepted, evidence-based framework that guides the design and evaluation of many dietary interventions—social cognitive theory (SCT) [31]. This paper describes the research design used to evaluate two theory-based interventions designed specifically to assist with the maintenance of a newly acquired dietary behavior (F&V consumption), one intervention guided by SCT and the other guided by GST.

## Methods

### Overview

**Figure 1:**
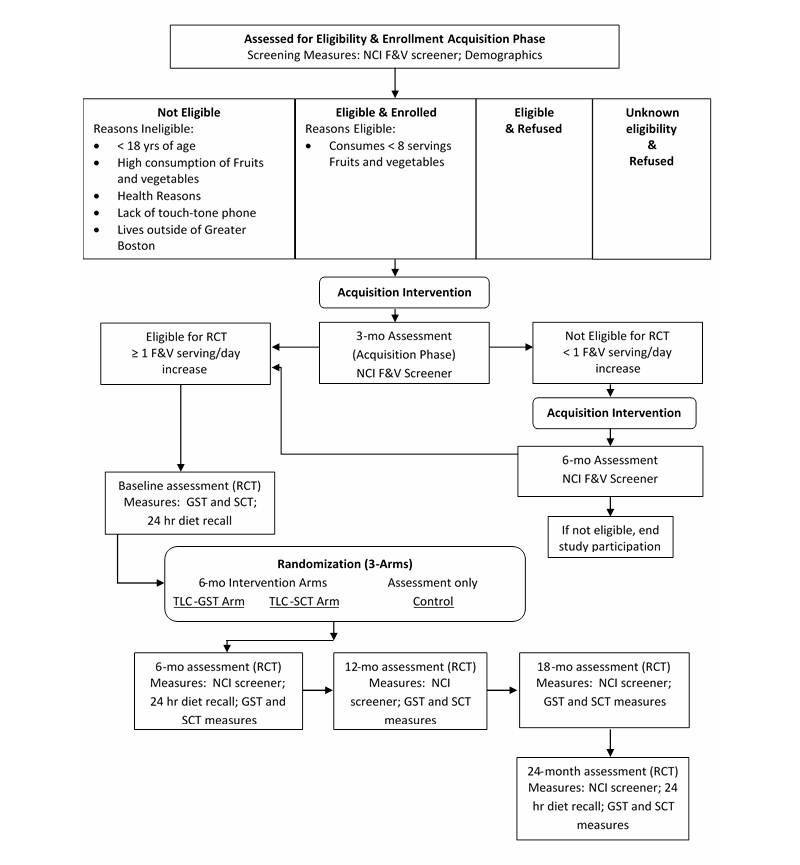
Flow diagram of the study.

The design for this study includes two phases to examine the maintenance of a dietary behavior change in adults. Figure 1 provides an overview of the study flow and measurement time points. In the first phase (acquisition), an intervention was used to produce a change in F&V consumption in order to study, in a second phase (maintenance), if an intervention targeting maintenance could sustain that change. All interventions were delivered using a fully automated telephony system that used an interactive voice response (IVR) system to generate speech that emulated counseling by a trained behavioral counselor combined with a speech recognition system to understand what the participant said. To perform the study, a sample of healthy adults who consumed less than the recommended level of F&V (ie, ≤ 5 servings/day) were recruited. During the acquisition phase, they were given a tested dietary telephony intervention for up to 6 months, called Telephone-Linked Care (TLC)-EAT, which has been shown in previous studies to have positive effects on initiating dietary improvements, including increased F&V consumption [32,33]. Because both prior studies found an increase of approximately one serving of F&V for the TLC-EAT intervention group, it was hypothesized that this effect would occur in a new sample of participants. F&V intake was examined at enrollment and after 3 months of the acquisition intervention. Participants who increased their consumption of F&V by at least one serving/day became eligible for the second phase (maintenance). Participants who did not show a one-serving increase were allowed to participate for an additional 3 months only. Those who achieved a one-serving increase during the acquisition study were eligible to participate in the second phase (maintenance, randomized controlled trial). Participants who entered the second (maintenance) phase of the study were blinded to the inclusion criterion (eg, an increase of >1 serving/day of F&V). Participants in the maintenance phase were randomized to one of three groups, receiving one of two theoretically based TLC interventions targeting maintenance of behavior (TLC-Maintenance GST or TLC-Maintenance SCT) or to the control group (an assessment only without an intervention). All participants in the randomized controlled trial (RCT) were assessed every 6 months for a total of 2 years post randomization (baseline). The study protocol received full board review and approval by the Boston University School of Medicine Institutional Review Board (NCT00148525). See Multimedia Appendix 1 for the CONSORT-EHEALTH checklist [34].

### Participants

The pool of possible participants was randomly selected from the voter registration lists from the Boston metropolitan area. Participants were required to be ≥18 years of age, live in the Boston area, have access to a touch-tone telephone, and be generally healthy. Participants were also required to be “under-consumers” of F&V as defined by eating fewer than 5 servings/day. However, in the first 2 months of recruitment, a large proportion (62%) reported consuming more than 5 servings/day using the National Cancer Institute F&V Screener (NCI FVS) [35]. Given that this measure may overestimate the servings of F&V [36-38], the inclusion criterion was modified to “consuming less than eight F&V servings/day” in order to correct for the bias in the NCI FVS. This modification decreased the percentage of screened individuals who were not eligible (39%) yet still captured adults who were not meeting a public health goal level, for example, the 2005 dietary guidelines of 9 servings/day of F&V [39-41]. Participants were not eligible if they were pregnant or if they had a recent health event such as a diagnosis of cancer, myocardial infarction, kidney disease, eating disorder, or were prescribed a special diet.

### Procedures

The University of Rhode Island Survey Research Center performed study recruitment, assessment, and randomization. The Center randomly selected names from a voter registration list and mailed a letter inviting them to participate in the study. The letter was followed by a phone call from the Center about one week later where the interviewer described the study, screened for eligibility (see Participant section above for eligibility criteria), obtained verbal consent, and administered study instruments. Research assistants at Boston University School of Medicine, where the intervention (ie, TLC-EAT) was hosted, sent participants an enrollment packet in the mail, which contained a welcome letter, consent form for their records, and a manual and personal password for the healthy eating acquisition intervention (ie, TLC-EAT). A week after enrollment, these research assistants called participants to train them on how to use TLC-EAT. Participants did a practice call with the research assistant on the line. Those who completed the training were transferred to the first intervention call (the “training call”) with TLC-EAT. Thereafter, all TLC calls were outbound calls initiated by the automated system, which called participants at the time initially entered into the automated scheduling system during the training call. Participants were asked to complete one TLC-EAT call per week for 12 weeks with the option of rescheduling any of the incoming TLC calls or initiating calls to TLC if preferred. After 12 weeks, F&V consumption was reassessed with the NCI FVS to determine eligibility for the maintenance phase of the trial. Participants who increased by one serving of fruit and/or vegetable were eligible to be randomized to one of three groups for the RCT. Random allocation to group assignment was generated by the SRC’s computer program that used urn randomization protocols to balance groups by gender. Those who did not increase by one serving after 3 months were invited to continue with the acquisition intervention, TLC-EAT, for an additional 3 months and then were assessed again for eligibility into the RCT. Those who qualified and agreed to continue in the RCT were assessed at baseline (randomization), 6, 12, 18 and 24 months post baseline. A random sample of those who qualified for the RCT were invited to complete a blood draw at the university’s General Clinical Research Center at baseline only. Assessments during the RCT included the NCI FVS, 24-hour dietary recalls, and psychosocial measures. Participants received US $20 for completing a survey at baseline, 6-month, 12-month, 18-month, and 24-month time points, and $50 for the in-person, fasting blood draw.

### Intervention

#### Overview

All interventions were delivered using a fully automated telephony system, TLC, which speaks to participants using computer-controlled, pre-recorded human speech, and the participant selects among pre-determined options to respond to the computer by either pressing keys on the telephone keypad or selecting an option by speaking into the phone [42]. TLC systems deliver an individualized intervention that mimics a conversation between a counselor and client.

#### Dietary Acquisition Intervention

The acquisition phase was delivered by TLC-EAT, designed to improve general diet quality by broadly targeting important nutrients in the diet, such as saturated fat and fiber, and by targeting food groups (eg, fruits, vegetables, whole grains), and by encouraging related dietary behaviors such as avoiding fried foods. See Multimedia Appendix 2, Table 1, for topics and previously published studies for details [32,33]. However, two modifications were made to TLC-EAT for the present study. The participants did not receive the two mailed printed reports received in previous trials. The second modification was that the present study used an “outbound” system (TLC contacts participant) to call participants on a weekly schedule instead of an “inbound” calling system (participant contacts TLC) used in the previous trials.

#### Maintenance Interventions

Two novel, theory-based interventions were used to promote the maintenance of dietary change with an emphasis on fruits and vegetables. Participants randomized to one of the two intervention arms of the RCT received ten automated TLC calls over the course of 6 months with the frequency of contacts reduced over time. These participants received one call per week in the first month (four calls in month one), one call every other week in the second month (two calls in month two), and one call per month for the remaining 4 months (one call in months three, four, five, and six). Calls were 10-15 minutes in duration. The first call after randomization was the same for both intervention groups and consisted of feedback on F&V, whole grains, low-fat dairy, and saturated and trans fats (see Table 2 in Multimedia Appendix 2). This call provided feedback for each of the food groups using data obtained from the Prime Screen [43], a screening instrument administered at the screening and randomization time points and used for intervention purposes only. Feedback consisted of progress made since the start of the acquisition intervention and comparisons to dietary recommendations. The remaining nine calls were different in content for the two intervention groups but not in duration (eg, 10-15 minutes).

#### Goal Systems Theory

GST posits that goals, as cognitive constructs, are mentally represented and organized and that this organization may help determine how goals are chosen and pursued [27-30]. This mental organization of goals into a system or structure assumes a hierarchy with primary goals and sub-goals. Primary goals are more abstract in nature yet have a large number of concrete means that represent specific behaviors used to attain that goal. GST also assumes that goals are linked to each other, and these inter-goal connections may play an important role in goal choice, goal pursuit, and goal attainment.

Maintaining a goal may depend on the characteristics of the goal and where that goal is within the goal system. A goal’s characteristics can include its perceived difficulty and value and its connection to other goals. Goals that are perceived as facilitating another goal are more likely to be pursued than goals that compete or conflict. Goals can be perceived as redundant or substitutable with other goals in one’s system because they fulfill the same underlying need or desire of another goal (eg, goal of exercising regularly to achieve health vs goal of healthful diet to achieve health). GST posits that the maintenance of goal pursuit will decrease the more the individual perceives the goal as substitutable or redundant with other pursuits. Thus, the greater the degree to which a goal is perceived to facilitate other goals while the less it is perceived to be redundant with these goals, the greater the likelihood its pursuit will be maintained. GST also suggests that the maintenance of pursuing a goal may be enhanced to the degree to which an individual integrates current pursuits with his or her other pursuits. GST-related research has found that integrating goals is associated with positive health outcomes [27,44-46].

#### Telephone-Linked Care Maintenance–Goal Systems Theory

GST was used to develop a TLC program that would query each TLC-Maintenance Goal Systems (TLC-GST) participant on how the person manages a diet goal (maintaining increased consumption of F&V) and other life goals. The general thesis is that maintenance of behavior change fails because of reasons outside of the specific behavioral domain, namely competition from other life goals. TLC-GST’s purpose was to train the participant in goal management techniques to help them maintain dietary changes. The four domains of goal management targeted in TLC-GST were (1) finding ways to reduce conflict between competing goals (ie, goal conflict reduction) [27], (2) applying strategies that use other goals to help attain diet goals (ie, cross goal facilitation [47]), (3) finding ways to protect or shield the targeted goal from other competing goals (ie, goal shielding [30]), and (4) enabling the individual to maintain the resources that are necessary for achievement of the targeted goal (eg, maintenance of increased F&V consumption) (ie, facilitating goal maintenance [48]).

TLC-GST used a cognitive-based approach in which participants were asked to think and reflect instead of the traditional skills-based approach generally used in SCT interventions. As described above, the first maintenance call was the same for both SCT and GST. The GST intervention began after this first call. The general structure of a TLC-GST counseling call began with a greeting, followed by counseling on a GST-related topic (see the section on Goal System Theory above), homework of suggested exercises, and a closing that included a reminder about the next call. In each of these calls, TLC-GST reminded the participants of their top four ranked life goals collected at randomization (see Table 3 in Multimedia Appendix 2) and asked them to select one to discuss on the call. The content of the conversation focused on a life goal that the participant chose, its attributes, and its relation to dietary (eg, F&V) goals. As mentioned earlier, homework was assigned at the end of each call (eg, “I’d like you to write down 3 of your larger goals. Then, think about how healthy eating can help you to meet those larger goals”); however, the strategy of goal setting, which was used in TLC-SCT, was not part of the TLC-GST intervention. See Table 2 in Multimedia Appendix 2 for more details on call structure and content.

#### Social Cognitive Theory

Within SCT, there are five constructs that are relevant to health interventions, namely knowledge, self-efficacy, outcome expectation, goal formation, and social-environmental factors [31]. The present maintenance study focused on increasing self-efficacy. Self-efficacy is related to whether a person will attempt a task and also to how long a person will persevere. Self-efficacy can be increased using strategies such as providing specific feedback, positive reinforcement, encouraging small steps towards a goal and goal setting. Evidence suggests that interventions designed to increase self-efficacy improve adherence to health behaviors [49-51], and it may be one of the strongest mediators of behavior change [52]. A health behavior model that draws from social cognitive theory, targets self-efficacy as a mediator of behavior, and focuses on maintaining a behavior change is the Relapse Prevention Model (RPM) [53]. A key strategy with RPM is the identification and anticipation of situations that can lead to relapse back to unhealthy behaviors. Planning ahead for these risky situations can increase the probability that an individual will be successful when faced with this challenge. When small successes are experienced, self-efficacy increases. Thus, in the present study the strategies of planning ahead to overcome or avoid risky situations were used to help maintain a newly acquired behavior and increase self-efficacy.

#### Telephone-Linked Care Maintenance–Social Cognitive Theory

The intervention was designed to assist with the maintenance of diet changes using intervention components to build self-efficacy. TLC-SCT was developed as an extension of the acquisition study intervention (TLC-EAT) with a focus on maintaining healthy eating rather than obtaining it. RPM [53] was used to inform what strategies should be included in a maintenance intervention, although the overarching goal for the ten calls was to remind participants about the skills they learned previously and continue to build knowledge and skills. The main skills included goal setting, identifying barriers to healthy eating, anticipating and planning ahead for situations that lead to unhealthy eating, and rewarding oneself for reaching goals. TLC addressed knowledge within the context of maintenance and provided specific, positive feedback on behaviors and goals obtained, an important component of building self-efficacy and mastery.

The dietary content for the ten TLC-SCT maintenance calls focused on F&V as well as the food groups targeted in TLC-EAT. The food group topics mirrored that of TLC-EAT because the initial changes to the individual’s F&V happened within the context of these food groups. Different from TLC-EAT, fruit and/or vegetable intake and goals were emphasized on every TLC-SCT call in addition to the food group topic. As mentioned previously, the first call for TLC-GST and TLC-SCT were the same. After the first call, the general structure of a call was (1) follow up on goals set previously, (2) assess intake of a food group and confidence to improve or maintain intake, (3) participant selects a barrier that impedes healthy eating and receives strategy for overcoming that barrier, (4) participant selects situation that tends to lead to relapse and hears tips for planning ahead, (5) participant given the option to set a goal for the main food group discussed and an option to set an additional fruit or vegetable goal, and (6) end with a take-away message about the call.

### Measures

#### Overview

Assessments for the acquisition phase included self-report questionnaires administered at the enrollment call, and 3 months and 6 months later. Measures included demographics, self-reported weight and height, and the NCI FVS. Additional measures were added to the maintenance phase (RCT) to more accurately estimate F&V outcomes and to examine change in theoretical constructs from SCT and GST. Baseline (randomization), 6 months, and 24 months were considered the primary time points for the RCT. Assessments at 12 and 18 months post baseline were considered secondary. A subsample of participants who were eligible for the RCT were randomly selected and invited to have a blood draw at baseline (randomization) to assess serum carotenoids. Other than the in-person blood draw, all assessments were completed over the telephone by survey research staff who were blinded to condition.

#### Fruit and Vegetable Intake

The primary outcome measure was F&V, and it was assessed two ways: brief screeners and 24-hour dietary recalls. The NCI FVS [35] was considered the primary screener and was administered at all assessment time points. The screener assesses self-reported frequency of consumption in the last month and the portion size for 10 items that included 100% fruit juice, fruit, lettuce salad, french fries or fried potatoes, white potatoes (not fried), cooked dried beans, other vegetables, tomato sauce, vegetable soup, and mixtures that include vegetables. Although all questions were asked, fried potato consumption and mixtures that include vegetables were not included in the summary scores or analyses. Validity of the FVS compared to true intake ranges from *r*=.66 for men to *r*=.51 for women [35].

For the primary assessment time points for the RCT (baseline, 6, and 24 months), the primary assessment of F&V was the NCI Method [54,55]. This method requires a food frequency assessment such as the NCI FVS and two 24-hour dietary recalls. The recalls were administered over the telephone by the Nutrition Epidemiology core at University of North Carolina, Chapel Hill, using the latest version of the Nutrition Data System for Research (NDSR) software. A nutritionist trained to conduct the telephone recalls used a standard introduction script and a multiple-pass approach interview methodology for the recall. The foods, beverages, preparation methods, amounts, and recipes reported by the participant were entered by an interviewer into the NDSR software to obtain an estimate of nutrient intake. The NDSR 2008 database was used for this study and contained over 18,000 foods, 8000 brand name products, many ethnic foods, supplements, and vitamins. The NDSR calculated food group serving count system was used to assess F&V.

Maintenance of F&V consumption was defined in this study as sustaining a one-serving/day increase in F&V achieved during the acquisition phase. Participants who increased their consumption by more than one serving during the acquisition phase need only maintain a one-serving gain to be considered as achieving maintenance. Maintenance (sustaining at least one serving/day) will be assessed at each time point, separately.

#### Carotenoid Levels

Participants who identified themselves as non-smokers were asked to fast for at least 6 hours prior to the blood draw. Samples were collected at a General Research Center under low light conditions and protected from light throughout processing [38]. The sera were stored at -70 degree C until analysis by a high-performance liquid chromatography method that was used to measure concentrations of alpha-carotene, beta-carotene, lycopene, lutein, zeaxanthin, and beta-cryptoxanthin, by the laboratory at Genox Corporation, Baltimore, MD.

#### Theoretical Constructs

Self-efficacy for F&V consumption was measured using a 6-item scale [56]. Participants were asked how confident they were about eating F&V in six different situations and responded on a 5-point scale: (1 not at all confident) and 5 (completely confident). GST constructs were assessed with a goal assessment battery that included five measures that were designed for this study because no scales existed. The battery included (1) identification of current life goals or priorities, (2) evaluation of success in meeting life goals, (3) evaluation of how current life goals or priorities previously selected conflict or facilitate healthy eating, (4) evaluation of life goal pursuit targeting the top life goal or priority that most interferes with eating a healthy diet, and (5) evaluation of dietary goal pursuit, respectively. First, participants were given a list of 15 life common goals (see Table 3 in Multimedia Appendix 2) and asked to identify those they were currently and actively trying to achieve (“goals or activities that you are spending time and effort on at least weekly”). The goals were ranked by the participants on a scale from 1-10 where 1 is not at all important and 10 is extremely important. Next, participants evaluated their success in meeting the goals by responding to the following question for each life goal: “How successful do you feel in meeting this goal right now?” Participants ranked each goal on a scale from 1-5 where 1 is not successful and 5 is extremely successful. Goal conflict and facilitation were measured by asking how the goals affect success in eating a healthy diet (“does the goal make it easier or harder for you to eat a healthy diet?”). The goal was ranked on a 5-point scale where 1 is much easier and 5 is much harder. An example of a question is “On a scale of 1 to 5, does getting more education or another degree make it easier or harder for you to eat a healthy diet?” Last, the construct of goal pursuit was assessed by asking the participant to evaluate the top life goal or priority that most interferes with eating a healthy diet. Participants selected the one priority or goal that interfered the most with eating a healthy diet, and they were asked to respond to a series of statements about pursing that goal (working toward this goal is exciting for me; I receive a lot of encouragement for working on this goal). Responses were on a scale where “1 is not at all true for me” and “5 describes me very well”. Goal pursuit for healthy eating was assessed last. Participants were asked to rate how well each of 29 statements described them: “I want you to think about the goal of eating a healthy diet. Please think about the goal of eating a healthy diet, and tell me how well each of these statements describes you. Please rate on a 1 to 5 scale where 1 is not at all true for you and 5 means it describes you very well.” Some sample items were “Working toward this goal is exciting”, “I try not to let other goals interfere with this goal”, and “I reward myself for working hard on this goal”.

#### Cost Assessment

Data were collected in order to complete a cost-effectiveness analysis of the maintenance interventions (see Data Analysis section). Direct costs were measured and included the TLC system costs (hardware, software, telephone, and labor). Development costs are excluded. The direct cost of implementing the interventions (TLC) was estimated by tracking the time a research assistant spent on tasks that would occur if the intervention were implemented outside of a research study (ie, labor costs of training personnel and operating and maintaining TLC). All tasks were categorized and tracked on the research assistants’ computers. Time spent on tasks that involved training the participant how to use TLC, telephone calls to assist the participant with TLC and computer server were tracked in a computer program. Research assistants logged onto their research operations system at the start of their workday, and any appropriate tasks were tracked by the system. Tasks that were specific to the research study were not tracked, such as assessment phone calls.

### Sample Size

Sample size for the RCT was based on 80% power to detect a 20% difference in the percentage maintaining an improvement in F&V consumption at 24 months. This sample size also provides 80% power to detect a small-to-medium effect size for differences in mean F&V consumption based on Cohen’s definition [57]. The primary hypothesis was that the treatment groups would maintain their initial gains in F&V consumption better than the control group, and the TLC-GST group would be significantly more successful in achieving this outcome than the TLC-SCT group (ie, GST > SCT > Control). This was tested defining maintenance as sustaining a one-serving increase in F&V that was achieved during the acquisition phase (as this is the eligibility criterion for the RCT). This primary hypothesis was tested using two approaches: a categorical approach and a continuous approach. The categorical hypothesis tests the proportion of the group who maintain, while the continuous tests the differences in F&V serving size. For the categorical hypothesis, the sample size estimation was based on a 20% difference in the proportion of participants meeting the definition of maintenance between groups. If the proportion maintaining gains is 80% in one group, 60% in another, and 40% in a third, there is 80% power of detecting pairwise differences between groups. Expecting 5% attrition at every 6 months from baseline to 24-month time point, a sample size of 405 (n=135 per group) is required at baseline and will yield a final sample of 330 (n=110 per group). This sample size provides 81% power of detecting the difference at 24-month time point between TLC-SCT and Control Groups (60% vs 40%), 87% power of detecting the difference between TLC-GST and TLC-SCT Groups (80% vs 60%), and 99% power of detecting the difference between TLC-GST and Control Groups (80% vs 40%), testing at the two-tailed alpha .05 level. For the continuous hypothesis, group differences are measured by the mean change from baseline to 24-month time point. A sample size of 110 provides 80% power of detecting an effect size of .38 of the standard deviation of the change score in F&V servings.

## Results

### Data Analysis

Data will be analyzed using SAS, version 9.1 for Windows. Alpha level was set at *P*<.05. Descriptive statistics will be used to characterize the different study subject samples before and after the acquisition study period. Descriptive statistics will examine those individuals who were screened for eligibility and those who qualified and enrolled into the RCT. Sample comparisons between those who were eligible and those who were not will be made using data collected at enrollment (screening data). Comparisons of the different study samples will be performed using Student’s *t* test for continuous variables and Pearson’s chi-square test for categorical variables. An additional analysis will be performed evaluating the change in F&V servings from initial screening (enrollment) to baseline (ie, the beginning of the maintenance phase) for the sample who was randomized into the RCT. An intention-to-treat approach using the last observation carried forward approach will be used to include those participants who drop out of the study or for whom there are missing data.

The primary hypotheses for the RCT is that the treatment groups will maintain their initial gains (ie, one-serving increase) in F&V consumption better than the control group, and the TLC-GST group will be significantly more successful in achieving this outcome than the TLC-SCT group (ie, GST > SCT > Control) on both a categorical (primary hypothesis) and a continuous measure (secondary hypothesis) of maintenance at all follow-up time points. The primary time points for the study will be baseline (randomization), 6 months (post intervention period), and 24 months (end of the follow-up period). The primary comparisons will be considered from baseline to 6 months, and baseline to 24 months. Group differences will be compared using a continuous variable (F&V consumption) at these time points. We will also compare groups on the percent who maintained a one-serving/day or greater increase in F&V consumption. The categorical measure of maintenance will be analyzed through multiple logistic regression models, and a continuous measure of maintenance will be analyzed through multiple linear regression models. Independent variables in these models will include a set of indicator variables for study group, baseline levels relating to each outcome variable, and potential confounders (variables found to differ between groups at baseline).

Longitudinal models will be used to explore group differences on dietary indicators over time, using data from all study evaluation points. These analyses will use the Generalized Estimating Equations (GEE) approach to accounting for the longitudinal nature of the data by modeling the within-subject correlation and adjusting both regression parameters and standard errors for this correlation. As compared to traditional repeated measures analysis of variance, the GEE approach allows for the inclusion of all available data from subjects with incomplete follow-up in the analysis. For categorical outcome measures, GEE logistic regression models for longitudinal data will be used, while for continuous outcome measures, GEE linear regression models will be used. Independent variables in these models will include a set of indicator variables for group, a set of indicator variables representing time (with baseline taken as the reference group), and a set of interaction terms modeling differential changes over time for the three study groups. Potential confounders identified in preliminary analyses will also be included in these models. Our primary interest is in the interaction terms, which will test whether the pattern of change in consumption of F&V over time differs by group.

While the primary focus of our analyses of the number of F&V servings is on changes from baseline to 6 to 24 months, our longitudinal models will also allow us to examine maintenance decay in the number of servings from 6 months (at the end of the maintenance intervention) to 24 months. Similarly, our longitudinal models for the categorized maintenance outcome will allow us to examine changes in maintenance from 6 to 24 months as well.

Secondary analyses will examine the influence of psychosocial variables. For TLC-SCT, we hypothesize that the outcomes are mediated by self-efficacy. For TLC-GST, we hypothesize that outcomes are at least partially explained by changes in goal system variables such as levels of inter-goal facilitation, inter-goal substitution, and inter-goal conflict at all major follow-up time points.

These hypotheses will be tested with path analysis. Potential mediational pathways of the effect between the randomized groups and F&V intake for the theoretical construct variables will be examined. Path models will be constructed to test the direct and indirect associations indicated by our research model. Using 6 and 12 month data as indicators of processes of maintenance in both the mid-term (at the end of the maintenance intervention) and in the longer term (6+ months post intervention), path analyses, both separate and combined, will be conducted to examine mediators of maintenance variables as influenced by group assignment. To examine the TLC-SCT change model, paths will be modeled from an intervention variable to self-efficacy to F&V intake. Direct and indirect effects of the intervention will be estimated through standardized path coefficients. For the TLC-GST, goal system variables will be examined in analogous path models.

### Cost Analysis

Cost analyses are planned for the study conditional upon demonstrating that the maintenance interventions are effective in altering and sustaining improvements in diet. The analysis will be based on the recommendations of the US Public Health Service Panel on Cost-Effectiveness Analysis [58]. An incremental cost-effectiveness ratio on the acquisition phase will be computed. The incremental cost-effectiveness of the two maintenance intervention conditions will be compared relative to the acquisition intervention to assess the resource use associated with incremental sustained improvements in health.

## Discussion

### Principal Considerations

There are a number of important issues to consider about this study. The overarching goal of this study is to better understand how to help individuals maintain a newly acquired health behavior. The challenge was first to identify a population that is engaging in unhealthful behaviors and that the behavior is amenable to change. F&V was selected as the principal target for the intervention study for multiple reasons. First, the majority of the US population is not consuming enough F&V, providing an opportunity to recruit a large enough sample within a reasonable time frame. Second, we had to identify an evidence-based, off-the-shelf intervention that would likely produce a change in F&V in order to study whether a newly acquired behavior can be sustained with interventions specific to the maintenance of that newly acquired behavior. While the TLC-EAT intervention was used for the acquisition phase of the study, we had to consider how much time it would take to achieve a primary intervention effect using TLC-EAT to make a meaningful positive dietary behavior change to qualify sufficient numbers of participants for the maintenance intervention RCT, and what should be considered a positive response in this RCT. One option was to offer TLC-EAT acquisition intervention for as long as it took the participant to show a positive improvement. This was not feasible given the time limitations of the study but fortunately was not a factor in the study design because the TLC-EAT acquisition intervention was known to achieve a positive effect within 3 months based on a previous study [31]. We also decided to define a positive intervention effect in the acquisition phase as an increase in F&V consumption by one or more servings per day. We did consider using a threshold criterion of a successful acquisition intervention effect of 5 servings a day but did not want to exclude individuals who might begin the acquisition phase at very low levels of F&V consumption and have a substantial acquisition intervention effect but still not meet the threshold of 5 servings of F&V/day. Moreover, including individuals who increase at least one F&V serving a day is in line with the usual level of change achieved in successful dietary change programs [6]. In addition, there is no evidence in the literature that increasing F&V consumption from 0-1 serving/day confers different risk reduction than going from 4-5 servings/day. Moreover, it is not known if it is easier to maintain a one-serving increase from 4-5 servings per day compared to a change from 0-1 servings per day, or whether a larger increase in F&V consumption (for example 2 or more servings/day) is more difficult to maintain than a smaller increase during behavior change acquisition.

A major consideration for our study was how to define maintenance. There is not a well-accepted definition of maintenance of a behavior change. It could be defined as a criterion outcome, which means that the individual consumes at least the same number of servings of F&V at each outcome measurement point as he or she consumed at the end of the acquisition phase. Another approach is to compare the three groups at each time point using the proportion of participants in each study group who reach criterion at that time (eg, consume at least the same number of servings of F&V at the end of acquisition to the end of the maintenance assessment). In doing so, we will consider the standard error of the measurement of F&V intake so that maintenance of F&V consumption will be defined as at least the same intake the person achieved at the end of the TLC-EAT acquisition period minus the standard error. Another approach is to compare changes in intake of F&V between randomization and each outcome measurement point. These “change scores” can be compared across the three study groups. The advantage of this approach over the criterion outcome approach is that it considers both the degree of preservation of intervention effect and possible increases in intervention effect by the maintenance intervention. Recent research suggests beneficial health effects as F&V consumption increases rather than only among those who meet a certain threshold (ie, 5 servings/day) [2]. The advantage of using a “change score” rather than a “threshold score” is that it may be the most relevant public health criterion. However, it is a less intuitive measure of maintenance than the criterion measure. Thus, both approaches will be examined.

The last consideration concerns the diet content of the basic TLC-EAT intervention. TLC-EAT has shown to improve F&V in two previous interventions yet the topics covered in the intervention calls include other food groups (diary, protein, grains). The approach taken in TLC-EAT relies heavily on the strategy of substitution of “healthy” for “unhealthy” foods; in doing so, it promotes greater consumption of some food groups, like F&V, possibly at the expense of others, like red and processed meats, and recognizes the public health benefits of targeting multiple dietary risk factors and the practical realities of intervening on diet. Thus, the diet topics in all of the interventions were written using this approach.

### Strengths

While few studies have evaluated maintenance-specific interventions for dietary change, fewer maintenance-specific interventions have been studied in which outcomes exceed 12 months, and even fewer have compared theory-guided interventions. The major innovation of this project is the design, creation, and testing of a maintenance-specific intervention (TLC-GST) and its comparison with an intervention based on the most commonly used behavioral theory, SCT. GST is a “maintenance-focused” theory in that it posits that many worthy and attainable goals fall to the wayside because other endeavors, for any number of reasons, take resources away from their pursuit. If it is competition for internal and external resources that triggers a significant proportion of relapse, then an intervention that successfully advocates for the continued allocation of resources to dietary behavior, as well as assists users in more efficiently managing the resources they have across their goals, should make a difference. It is also possible that maintenance of goal behaviors that do not have immediate and perceivable rewards, especially true of long-term risk reduction, is especially appropriate for an intervention that helps balance demands from goals that have more short-term and palpable rewards.

Few studies have compared theories, mostly because it is a challenging task that requires that at least two interventions be available that are the same or very similar with each of them based on a different behavioral theory. There are very few pairs of behavioral interventions that have functional comparability while being driven by distinctly different theories. Practically speaking, a researcher has to design the two interventions being studied, and this is time-consuming and expensive. Nonetheless, this is exactly what we did in creating two theoretically guided interventions for maintenance of dietary behavior change. Using SCT to map out a maintenance intervention was doable because it is one of the most widely used theories for changing and maintaining health behaviors. The relapse prevention model [53], which is informed by SCT, provided a practical framework for implementing a SCT-based behavior maintenance intervention by explicitly identifying which intervention strategies to include (eg, goal setting, planning ahead) to maintain a behavior change. The resulting TLC-Maintenance SCT intervention has good fidelity to SCT. In contrast, and to our knowledge, GST has never been applied to health behavior change, whereas it has a strong theoretical and experimental underpinning for application to health behavior and its change. No study has attempted to translate and apply GST for practical dietary interventions delivered to individuals over the telephone.

Another strength of the study design in our RCT is our ability to control for fidelity in delivering the interventions. The acquisition intervention, TLC-EAT, was delivered to all enrolled participants in the same way because it was delivered using an automated computer system. TLC-EAT is designed to provide a consistent intervention of all users that is tailored to the participant. TLC-Maintenance SCT also provides a consistent maintenance intervention for all of its users. Although a computer-controlled health behavior change intervention may or may not be as tailored as a human counselor intervention, the intervention content is explicit and discoverable and has complete fidelity. If differences are found between the two health behavior change maintenance interventions, they will be due to the underlying content of the interventions and not due to differences in provider training, provider skill level, or lack of adherence to protocol [59].

### Conclusions

This study used a novel study design to initiate and then promote the maintenance of dietary behavior change through the use of an evidence-based fully automated telephony intervention. After the first 6 months (the acquisition phase), we will examine whether two telephony interventions built using different underlying behavioral theories, were more successful than an assessment-only control group in helping participants maintain their newly acquired health behavior change.

## Multimedia Appendix 1

CONSORT-EHEALTH checklist V1.6.2 [59].

## Multimedia Appendix 2

Supplementary tables.

## Abbreviations

F&V: fruits and vegetables
GST: Goal Systems Theory
IVR: interactive voice response
NCI FVS: National Cancer Institute Fruit and Vegetable Screener
RCT: randomized controlled trial
RPM: relapse prevention model
SCT: Social Cognitive Theory
SRC: University of Rhode Island’s Survey Research Center
TLC: telephone-linked care
TLC-EAT: telephone-linked care delivered dietary acquisition intervention
TLC-GST: telephone-linked care delivered dietary maintenance intervention based on Goal Systems Theory
TLC-SCT: telephone-linked care delivered dietary maintenance intervention based on Social Cognitive Theory
