# Supplementary material for: Comparison of Two Theory-Based, Fully Automated Telephone Interventions Designed to Maintain Dietary Change in Healthy Adults: Study Protocol of a Three-Arm Randomized Controlled Trial
Source: JMIR Res Protoc. 2014 Nov 10;3(4):e62. doi: 10.2196/resprot.3367 (PMC4260007; doi:10.2196/resprot.3367)
Supplement: Supplementary file 1 [file resprot_v3i4e62_app1.pdf]

CONSORT-EHEALTH Checklist V1.6.2 Report

(based on CONSORT-EHEALTH V1.6), available at [http://tinyurl.com/consort-ehealth-v1-6].

3367

Date completed

5/24/2014 11:56:03

by

Julie Wright

The comparison of two theory-based, fully automated telephone interventions designed to maintain a recently acquired dietary change in healthy adults: Study protocol of a three-arm randomized controlled trial

TITLE

1a-i) Identify the mode of delivery in the title

"fully automated telephone interventions"

1a-ii) Non-web-based components or important co-interventions in title

N/A. We did not have a co-intervention method/channel.

1a-iii) Primary condition or target group in the title

We do not include this information in the title because our title is lengthy. But, we do state in the abstract, "a sample of adults from a large urban."

ABSTRACT

1b-i) Key features/functionalities/components of the intervention and comparator in the METHODS section of the ABSTRACT

"This study compared two theory-based interventions (Social Cognitive Theory (SCT) v. Goal Systems Theory (GST)) designed to maintain previously achieved improvements in fruit and vegetable (F&V) consumption. The interventions used tailored, interactive conversations delivered by a fully automated telephony system [Telephone Linked Care (TLC)] over a 6-mo period."

1b-ii) Level of human involvement in the METHODS section of the ABSTRACT

"The interventions used tailored, interactive conversations delivered by a fully automated telephony system [Telephone Linked Care (TLC)] over a 6-mo period."

1b-iii) Open vs. closed, web-based (self-assessment) vs. face-to-face assessments in the METHODS section of the ABSTRACT

"All participants who qualified for the RCT completed surveys administered by blinded interviewers at baseline..."

1b-iv) RESULTS section in abstract must contain use data

N/A to a research protocol paper.

1b-v) CONCLUSIONS/DISCUSSION in abstract for negative trials

N/A. This manuscript does not report results.

INTRODUCTION

2a-i) Problem and the type of system/solution

Problem: "In recent years, multiple commentators have called for the need to sustain short term health behavioral intervention effects by studying intervention effects at the end of the intervention period as well as long term follow-up after the intervention concluded, and "maintenance of effects for vegetable intake was not sustained during the periods of no intervention contact despite initial improvements immediately following the interventions" and. And, "The present study conceptualizes maintenance as a distinct phase of the health behavior change process, and proposes Goal Systems Theory (GST) [26–29] as a possible theory to inform the design of a maintenance intervention to assist individuals with managing their newly acquired behavior (increased consumption of F&V) following the acquisition of this behavior. This novel approach will be compared to a widely accepted, evidence-based framework that guides the design and evaluation of many dietary interventions, social cognitive theory (SCT).[30] This paper describes the research design used to evaluate two, theory-based interventions designed specifically to assist with the maintenance of a newly acquired dietary behavior (F&V), one being guided by SCT and the other being guided by GST."

Type of system: "All interventions were delivered using a fully automated telephony system, TLC, which speaks to participants using computer-controlled, pre-recorded human speech, and the participant selects among pre-determined options to respond to the computer by either pressing keys on the telephone keypad or selecting an option by speaking into the phone.[40] TLC systems deliver an individualized intervention that mimics a conversation between a counselor and client."

2a-ii) Scientific background, rationale: What is known about the (type of) system

We do not describe the rationale for the system in the Introduction because comparing the system to other channels was not the main point of this study. The system was a fully automated telephony intervention which has been used in numerous trials. The reason this study used TLC was that it provided an evidence-based program that we needed to compare the two theories.

METHODS

3a) CONSORT: Description of trial design (such as parallel, factorial) including allocation ratio

The hypotheses are stated in the Abstract and the Data analysis section but not in the introduction.

3b) CONSORT: Important changes to methods after trial commencement (such as eligibility criteria), with reasons

We included the changes after trial commencement.

"Participants were also required to be “under-consumers” of F&V as defined by eating fewer than five servings/day. However, in the first two months of recruitment, a large proportion (62%) of those contacted were reporting more than five servings/d using the National Cancer Institute F&V Screener (NCI FVS). [33] Given that this measure may overestimate the servings of F&V,[34–36] the inclusion criterion was modified to “consuming less than eight F&V servings/day” in order to correct for the bias in the NCI FVS. This modification decreased the percentage not eligible (39%) yet still captured adults who were not meeting the 2005 dietary guidelines, i.e., 9 servings of F&V a day.[37–39] "

3b-i) Bug fixes, Downtimes, Content Changes

During the acquisition phase, as with all computer systems, the server crashed but a back up server was on-line the same day or by the next day. The system was an "outbound" system that called/contacted participants. If the server went down, a call was delayed by a day. The call scheduled was 1 call per week but was not designed to be absolutely fixed, i.e., must be 7 days apart. We anticipated that participants might get behind or miss a call because of life events. These events did not change the study design either.

4a) CONSORT: Eligibility criteria for participants

To be eligible for the acquisition/run-in phase: "Participants were required to be >18 years of age, live in the Boston area, have access to a touch tone telephone, and be generally healthy. Participants were also required to be “under-consumers” of F&V as defined by eating fewer than five servings/day."

To be eligible for the RCT, "Those who achieved a one serving increase during the acquisition study were eligible to participate in the second phase (the RCT)."

4a-i) Computer / Internet literacy
The telephone system could be used on a land-line or cell phone.

" A week after enrollment, a research assistant called participants to train them on how to use TLC-EAT. Participants did a practice call with the research assistant on the line. Those who completed the training were transferred to the first intervention call (the “training call”) with TLC-EAT."

4a-ii) Open vs. closed, web-based vs. face-to-face assessments:

"Other than the in-person blood draw, all assessments were completed over the telephone by survey research staff who were blinded to condition."

"The recalls were administered over the telephone by the Nutrition Epidemiology core at UNC-CH using the latest version of the Nutrition Data System for Research (NDSR) software (www.ncc.umn.edu). A nutritionist trained to conduct the telephone recalls used a standard introduction script and a multiple-pass approach interview methodology for the recall."

4a-iii) Information giving during recruitment
"A survey research center (SRC) located at the University of Rhode Island was used for recruitment, assessment and randomization. The center randomly selected names from a voter registration list and mailed a letter inviting them to participate in the study. The letter was followed by a phone call from the center about one week later where the caller described the study, obtained verbal consent, and administered study instruments. Research assistants located at Boston University School of Medicine, where the intervention (i.e., TLC-EAT) was hosted, sent participants an enrollment packet in the mail which contained a welcome letter, consent form, and a manual and personal password for the healthy eating acquisition intervention (i.e., TLC-EAT). A week after enrollment, these research assistants called participants to train them on how to use TLC-EAT."

4b) CONSORT: Settings and locations where the data were collected
"A survey research center (SRC) located at the University of Rhode Island was used for recruitment, assessment and randomization."

"Research assistants located at Boston University School of Medicine, where the intervention (i.e., TLC-EAT) was hosted, sent participants an enrollment packet in the mail which contained a welcome letter, consent form, and a manual and personal password for the healthy eating acquisition intervention (i.e., TLC-EAT). A week after enrollment, these research assistants called participants to train them on how to use TLC-EAT."

"Other than the in-person blood draw, all assessments were completed over
the phone"

"The recalls were administered over the telephone by the Nutrition Epidemiology core at UNC-CH using the latest version of the Nutrition Data System for Research (NDSR) software."

4b-i) Report if outcomes were (self-)assessed through online questionnaires
N/A. The study used survey center staff to administer all surveys.

"Other than the in-person blood draw, all assessments were completed over the telephone by survey research staff who were blinded to condition."

4b-ii) Report how institutional affiliations are displayed
We did not report in the manuscript. The recruitment letter and consent form used the university's logo.

5) CONSORT: Describe the interventions for each group with sufficient details to allow replication, including how and when they were actually administered

5-i) Mention names, credential, affiliations of the developers, sponsors, and owners
N/A. Other than the recruitment letter and consent form including the logo of the university, we did not mention names, credentials, etc. We did not have a conflict of interest to declare.

5-ii) Describe the history/development process
"They were given a tested dietary telephony intervention for up to 6 months, called Telephone-Linked Care (TLC)-EAT, which we have shown to have positive effects on diet, including F&V, in two previous trials. [31,32] "

5-iii) Revisions and updating
N/A. The intervention delivered by TLC was "frozen" during the trial. There was no dynamic content.

5-iv) Quality assurance methods
N/A.

5-v) Ensure replicability by publishing the source code, and/or providing screenshots/screen-capture video, and/or providing flowcharts of the algorithms used
Source code is N/A for this system. The Methods section and Tables presented describe detailed information on the content of the intervention. The content of the intervention consists of scripts or dialog. These can be delivered to other researchers who want to replicate the study.

5-vi) Digital preservation
N/A. The intervention is a telephony system.

5-vii) Access
"Eligible participants who agreed to participate were sent an enrollment packet in the mail which contained a welcome letter, consent form, and a manual and personal password for the healthy eating acquisition intervention (i.e., TLC-EAT)."

"Participants received $20 US dollars for completing a survey at baseline, 6-mo, 12-mo, 18-mo, and 24-mo time points, and $50 for the in-person, fasting blood draw."

5-viii) Mode of delivery, features/functionalities/components of the intervention and comparator, and the theoretical framework

The paper goes into extensive detail on the mode of delivery, features and theoretical framework. For example:

"All interventions were delivered using a fully automated telephony system, TLC, which speaks to participants using computer-controlled, pre-recorded human speech, and the participant selects among pre-determined options to respond to the computer by either pressing keys on the telephone keypad or selecting an option by speaking into the phone.[40] TLC systems deliver an individualized intervention that mimics a conversation between a counselor and client."

"TLC-Maintenance GST. GST was used to develop a TLC program that would query each TLC-Maintenance Goal Systems (TLC-GST) participant on how the person manages a diet goal (maintaining increased consumption of F&V) and other life goals. The general thesis is that maintenance of behavior change fails because of reasons outside of the specific behavioral domain, namely competition from other life goals..."

"Social Cognitive Theory. Within SCT, there are five constructs which are relevant to health interventions, namely knowledge, self-efficacy, outcome expectation, goal formation, and social-environmental factors.[30] The present maintenance study focused on increasing self-efficacy. Self-efficacy is related to whether a person will attempt a task and also to how long a person will persevere. Self-efficacy can be increased using strategies such as providing specific feedback, positive reinforcement, encouraging small steps towards a goal and goal setting..."

5-ix) Describe use parameters
"Participants were asked to complete one TLC-EAT call per week for 12 weeks with the option of rescheduling any of the incoming TLC calls or initiating calls to TLC if preferred."

"These participants received one call per week in the first month (four calls in month one), one call every other week in the second month (two calls in month two), and one call per month for the remaining four months (one call in months three, four, five and six). Calls were 10-15 minutes in duration."
5-x) Clarify the level of human involvement
All interventions were delivered using a fully automated telephony system sometimes described as interactive voice response (IVR) system.

"Those who completed the training were transferred to the first intervention call (the “training call”) with TLC-EAT. Thereafter, all TLC calls were outbound calls initiated by the automated system which called participants at the time initially entered into the automated scheduling system during the training call. Participants were asked to complete one TLC-EAT call per week for 12 weeks with the option of rescheduling any of the incoming TLC calls or initiating calls to TLC if preferred."
5-xi) Report any prompts/reminders used
"Those who completed the training were transferred to the first intervention call (the “training call”) with TLC-EAT. Thereafter, all TLC calls were outbound calls initiated by the automated system which called participants at the time initially entered into the automated scheduling system during the training call. Participants were asked to complete one TLC-EAT call per week for 12 weeks with the option of rescheduling any of the incoming TLC calls or initiating calls to TLC if preferred."
5-xii) Describe any co-interventions (incl. training/support)
N/A. This study did not include a co-intervention.

"Those who completed the training were transferred to the first intervention call (the “training call”) with TLC-EAT. Thereafter, all TLC calls were outbound calls initiated by the automated system which called participants at the time initially entered into the automated scheduling system during the training call. Participants were asked to complete one TLC-EAT call per week for 12 weeks with the option of rescheduling any of the incoming TLC calls or initiating calls to TLC if preferred."
6a) CONSORT: Completely defined pre-specified primary and secondary outcome measures, including how and when they were assessed
"F&V Intake. The primary outcome measure was F&V, and it was assessed two ways: brief screeners and 24-hour dietary recalls. The NCI Fruit and Vegetable Screener (NCI FVS) [33] was considered the primary screener and was administered at all assessment time points."

"Secondary analyses will examine the influence of psychosocial variables. For TLC-SCT, we hypothesize that the outcomes are mediated by self-efficacy. For TLC-GST, we hypothesize that outcomes are at least partially explained by changes in goal system variables such as levels of inter-goal facilitation, inter-goal substitution, and inter-goal conflict at all major follow-up time points."
6a-i) Online questionnaires: describe if they were validated for online use and apply CHERRIES items to describe how the questionnaires were designed/deployed
N/A. We did not use on-line questionnaires.
6a-ii) Describe whether and how “use” (including intensity of use/dosage) was defined/measured/monitored
N/A. We did not include this information in the protocol paper. Dose was not considered a variable in our data analysis.
6a-iii) Describe whether, how, and when qualitative feedback from participants was obtained
We did not include this in the protocol paper.
6b) CONSORT: Any changes to trial outcomes after the trial commenced, with reasons
N/A. The manuscript is a protocol paper, no results are presented.
7a) CONSORT: How sample size was determined
7a-i) Describe whether and how expected attrition was taken into account when calculating the sample size

7b) CONSORT: When applicable, explanation of any interim analyses and stopping guidelines
N/A. This study examines maintenance of effects over 24 months.
8a) CONSORT: Method used to generate the random allocation sequence
"Random allocation to group assignment was generated by the SRC’s computer program that used urn randomization protocols to balance groups by gender."
8b) CONSORT: Type of randomisation; details of any restriction (such as blocking and block size)
"Random allocation to group assignment was generated by the SRC’s computer program that used urn randomization protocols to balance groups by gender."
9) CONSORT: Mechanism used to implement the random allocation sequence (such as sequentially numbered containers), describing any steps taken to conceal the sequence until interventions were assigned
"Random allocation to group assignment was generated by the SRC’s computer program that used urn randomization protocols to balance groups by gender.."
10) CONSORT: Who generated the random allocation sequence, who enrolled participants, and who assigned participants to interventions
"Random allocation to group assignment was generated by the SRC’s computer program that used urn randomization protocols to balance groups by gender."

11a) CONSORT: Blinding - If done, who was blinded after assignment to interventions (for example, participants, care providers, those assessing outcomes) and how
11a-i) Specify who was blinded, and who wasn't
Research assessors were blinded to condition. The assessors were located at a separate university from the study investigators.

"Other than the in-person blood draw, all assessments were completed over the telephone by survey research staff who were blinded to condition."

"The [diet] recalls were administered over the telephone by the Nutrition Epidemiology core at UNC-CH."
11a-ii) Discuss e.g., whether participants knew which intervention was the "intervention of interest" and which one was the "comparator"
This is not discussed in the manuscript. Participants in the assessment only group could have figured out that they were not getting as much attention as those in the other two conditions by reading the consent form. The other two conditions both received TLC calls but given that they were delivered to the participants at home, the risk of contamination was slim.
11b) CONSORT: If relevant, description of the similarity of interventions
Tables 1-3 describe the interventions.
12a) CONSORT: Statistical methods used to compare groups for primary and secondary outcomes
"Longitudinal models will be used to explore group differences on dietary indicators over time, using data from all study evaluation points. These analyses will use the Generalized Estimating Equations (GEE) approach to accounting for the longitudinal nature of the data by modeling the within-subject correlation and adjusting both regression parameters and standard errors for this correlation. As compared to traditional repeated measures analysis of variance, the GEE approach allows for the inclusion of all available data from subjects with incomplete follow-up in the analysis. For categorical outcome measures, GEE logistic regression models for longitudinal data will be used, while for continuous outcome measures, GEE linear regression models will be used. Independent variables in these models will include a set of indicator variables for group, a set of indicator variables representing time (with baseline taken as the reference group), and a set of interaction terms modeling differential changes over time for the three study groups."
12a-i) Imputation techniques to deal with attrition / missing values
"An intention-to-treat approach using the last observation carried forward approach will be used to include those participants who drop out of the study or for whom there are missing data."
12b) CONSORT: Methods for additional analyses, such as subgroup analyses and adjusted analyses
"These [secondary] hypotheses will be tested with path analysis. Potential mediational pathways of the effect between the randomized groups and F&V intake for the theoretical construct variables will be examined. Path models will be constructed to test the direct and indirect associations indicated by our research model. Using 6 and 12 month data as indicators of processes of maintenance in both the mid-term (at the end of the maintenance intervention) and in the longer term (6+ months post intervention), path analyses, both separate and combined, will be conducted to examine mediators of maintenance variables as influenced by group assignment. To examine the TLC-SCT change model, paths will be modeled from an intervention variable to self-efficacy to F&V intake. Direct and indirect effects of the intervention will be estimated through standardized path coefficients. For the TLC-GST, goal system variables will be examined in analogous path models."

"Cost Analysis. Cost analyses are planned for the study conditional upon demonstrating that the maintenance interventions are effective in altering and sustaining improvements in diet. The analysis will be based on the recommendations of the PHS Panel on Cost-Effectiveness Analysis.[55] An incremental cost-effectiveness ratio on the acquisition phase will be computed. The incremental cost-effectiveness of the two maintenance intervention conditions will be compared relative to the acquisition intervention to assess the resource use associated with incremental sustained improvements in health."

RESULTS

13a) CONSORT: For each group, the numbers of participants who were randomly assigned, received intended treatment, and were analysed for the primary outcome
N/A. The manuscript is a description of the protocol.
13b) CONSORT: For each group, losses and exclusions after randomisation, together with reasons
N/A. The manuscript is a description of the protocol.
13b-i) Attrition diagram
N/A. The manuscript is a description of the protocol.
14a) CONSORT: Dates defining the periods of recruitment and follow-up
N/A. The manuscript is a description of the protocol.
14a-i) Indicate if critical "secular events" fell into the study period
N/A. The manuscript is a description of the protocol.
14b) CONSORT: Why the trial ended or was stopped (early)
N/A. The manuscript is a description of the protocol.
15) CONSORT: A table showing baseline demographic and clinical characteristics for each group
N/A. The manuscript is a description of the protocol.
15-i) Report demographics associated with digital divide issues
N/A. The manuscript is a description of the protocol.
16a) CONSORT: For each group, number of participants (denominator) included in each analysis and whether the analysis was by original assigned groups
16-i) Report multiple "denominators" and provide definitions
N/A. The manuscript is a description of the protocol.
16-ii) Primary analysis should be intent-to-treat
N/A. The manuscript is a description of the protocol.
17a) CONSORT: For each primary and secondary outcome, results for each group, and the estimated effect size and its precision (such as 95% confidence interval)
N/A. The manuscript is a description of the protocol.
17a-i) Presentation of process outcomes such as metrics of use and intensity of use
N/A. The manuscript is a description of the protocol.
17b) CONSORT: For binary outcomes, presentation of both absolute and relative effect sizes is recommended
N/A. The manuscript is a description of the protocol.
18) CONSORT: Results of any other analyses performed, including subgroup analyses and adjusted analyses, distinguishing pre-specified from exploratory
N/A. The manuscript is a description of the protocol.

18-i) Subgroup analysis of comparing only users
N/A. The manuscript is a description of the protocol.
19) CONSORT: All important harms or unintended effects in each group
N/A. The manuscript is a description of the protocol.
19-i) Include privacy breaches, technical problems
N/A. The manuscript is a description of the protocol.
19-ii) Include qualitative feedback from participants or observations from staff/researchers
N/A. The manuscript is a description of the protocol.

DISCUSSION

20) CONSORT: Trial limitations, addressing sources of potential bias, imprecision, multiplicity of analyses
20-i) Typical limitations in ehealth trials
N/A. The manuscript is a description of the protocol.
21) CONSORT: Generalisability (external validity, applicability) of the trial findings
21-i) Generalizability to other populations
N/A. The manuscript is a description of the protocol.
21-ii) Discuss if there were elements in the RCT that would be different in a routine application setting
N/A. The manuscript is a description of the protocol.
22) CONSORT: Interpretation consistent with results, balancing benefits and harms, and considering other relevant evidence
22-i) Restate study questions and summarize the answers suggested by the data, starting with primary outcomes and process outcomes (use)
N/A. The manuscript is a description of the protocol.
22-ii) Highlight unanswered new questions, suggest future research
N/A. The manuscript is a description of the protocol.

Other information

23) CONSORT: Registration number and name of trial registry
Trials#NCT00148525
24) CONSORT: Where the full trial protocol can be accessed, if available
N/A. The manuscript is a description of the protocol.
25) CONSORT: Sources of funding and other support (such as supply of drugs), role of funders
"This study was funded by a grant R01CA105832"
X26-i) Comment on ethics committee approval
"The study protocol received full-board review and approval by the Boston University School of Medicine Institutional Review Board."
x26-ii) Outline informed consent procedures
"The letter was followed by a phone call from the center about one week later where the caller described the study, obtained verbal consent, and administered study instruments. Research assistants located at Boston University School of Medicine, where the intervention (i.e., TLC-EAT) was hosted, sent participants an enrollment packet in the mail which contained a welcome letter, consent form..."
X26-iii) Safety and security procedures
No, ensuring safety and security is in the IRB protocol/approval. This information is not necessary in the manuscript.
X27-i) State the relation of the study team towards the system being evaluated
"The authors declare that they have no competing interests."
