# Supplementary material for: Comparison of Two Theory-Based, Fully Automated Telephone Interventions Designed to Maintain Dietary Change in Healthy Adults: Study Protocol of a Three-Arm Randomized Controlled Trial
Source: JMIR Res Protoc. 2014 Nov 10;3(4):e62. doi: 10.2196/resprot.3367 (PMC4260007; doi:10.2196/resprot.3367)
Supplement: Supplementary file 2 [file resprot_v3i4e62_app2.pdf]

Table 1. Acquisition intervention (TLC-EAT) topics by week

TLC-EAT	Call Topics
Week 0	Training and practice with TLC while research assistant on the line. TLC provides feedback on Prime screen after research assistant hangs up.
Week 1	Main topic: Fruit. Quiz on fruits; Set Fruit goal
Week 2	Main topic: Meat, fish, poultry group. Recap vegetarian and lean protein; follow-up on fruit goals
Week 3	Main topic: Vegetables. Quiz vegetables; Vegetable goal; Recap vegetable message
Week 4	Main topic: Snacks, sweets, desserts. Quiz: Sweets/snacks; follow-up on Veg goal; Motivate to eat less fatty meats; Recap healthy desserts
Week 5	Main topic: Grain group. Dine out: Mexican; Quiz: starches; Recap whole grains
Week 6	Main topic: Diary group. Quiz dairy. Talk about Shopping meat, fish, etc. Recap low fat dairy
Week 7	How are we doing? Check-in
Week 8	Main topic: Beverages. Talk about Dining: Italian; Recap Beverages
Week 9	Main topic: Fats, spreads, oils. Query oils & spreads; Dine out, Chinese; Quiz on fattening foods; Recap "good" fat
Week 10	Main topic: Fruits. Re-query: fruit intake and provide feedback: Fruits. Set fruit goal.
Week 11	Re-query meat/fish poultry; follow-up on fruits; Recap lean protein message
Week 12	Re-query vegetables; New quiz vegetables; Provide feedback on vegetables

Table 2. Outline for the 10 TLC calls for the theory-based maintenance interventions

Timing	TLC-SCT Intervention	TLC-GST Intervention
Mo. 1 Calls 1-4	1. Congratulations on improvements made to fruit and/or vegetable servings<br>2. Description of maintenance calls for the next 6 months<br>3. Results of the PrimeScreen survey (fruit, vegetable, whole grains, low-fat dairy, saturated and trans fat) and how current intake compares to dietary recommendations and changes from the first Prime Screen administered for TLC-EAT<br>4. Closing: Reminder about next call in one week	First call is the same as the TLC-SCT call.
	1. Follow-up on last week’s goal	1. GST principles introduced:

2. Assess: Vegetable intake and suggest a challenge to increase vegetables at lunch time<br>3. Main topic is fruit: a) Barrier assessment (time, cost, availability, prep, spoilage, taste, fear of calories); b) Anticipating barriers and planning ahead to avoid them<br>4. Goal setting: Fruit<br>5. Closing: Remind about goals/challenges set and take-away message about fruit	Goal conflict and goal facilitation.<br>2. Resource management (How your goals can compete for the same personal resources)<br>3. Explore why another goal conflicts with diet goals with topics limited to time, physical energy, mental energy, and money
1. Follow-up on goal of eating vegetables at lunch time<br>2. Assess: When fruit is consumed<br>3. Main topic is protein/lean meat a) Barrier assessment (Lack of time; Lack of money; healthy protein costs too much; No places to buy healthy protein options; Don’t know how to prepare; Spoils too fast; Not in the habit; Don’t like taste; Family doesn’t like it), b) Planning ahead to overcome possible barriers<br>4. Follow-up on main goal of eating fruit<br>5. Goal setting: Protein and/or fruit and/or vegetable<br>6. Closing: Remind about goals/challenges set and take-away message about call topics	1. Introducing GST principle: Goal shielding.<br>2. Discuss prioritization of goals<br>a) Reflection on personal goals and priorities<br>b) Reasons to eat a healthful diet and uniqueness of benefits<br>3. Vignettes with Jack for male participants, and Jill for female participants
1. Follow-up on fruit goal from last week<br>2. Brief educational quiz on fruit recommendations<br>3. Assess: Fruit shopping habits with feedback and suggestions for improvement<br>4. Main topic is vegetables: a) Barrier assessment (Lack of time, money, No place to buy fresh vegetables, Don’t know how to prepare them, They spoil too fast, Not in the habit of eating vegetables, Don’t like taste of them); b)	1. GST principle: Goal shielding continued<br>2. Temptations (avoiding overload, simplifying life)<br>3. Planning for the unexpected

	Planning ahead to overcome barriers<br>5. Goal setting: Vegetable goal<br>6. Closing: Take home reminder about F&V	
Mo. 2<br>Calls<br>5-6	1. Follow-up of vegetable goal<br>2. Assess: When vegetables are eaten and feedback and suggestions<br>3. Main topic is sweets: a) brief assessment on how much you are eating, b) confidence in limiting, c) barrier assessment (lack of time to make healthy snacks, Lack of money to buy healthy foods, No place to buy healthy foods at work, Craving for sweets is strong, Eating sweets is a habit, Children like them so I keep them in the house, They’re always available at work, They’re hard to resist at parties and friends’ houses) d) Planning ahead to overcome barriers<br>4. Goal setting: Sweets<br>5. Closing: take away and reminder about next call	1. Review top 4 most important goals that participant selected at baseline, participant selects one life goal to discuss<br>2. Determine how the goal affects a goal of eating healthy (easier, harder, neither)<br>3. Provide diet tips to help with highest ranking resource management issue (time, money, physical energy, mental energy).
	1. Follow-up on veg and/or sweets goal<br>2. Assess: assessment of fruit and feedback – and ask to add fruit for a small goal<br>3. Main topic is whole grains: a) brief assessment on how much you are eating, b) confidence in limiting, c) barrier assessment (lack of time, etc.) d) Planning ahead to overcome barriers<br>4. Goal setting: grains and/or fruit<br>5. Closing: take away and reminder about fruit goal	1. GST principle: Goal redundancy discussed.<br>2. Discuss how to decrease perceived redundancy in goals (e.g. how to use smaller goals to work toward reaching a larger goal)
Mo. 3<br>Call 7	1. Follow-up on grains and/or fruit<br>2. Assess: assessment of vegetable intake, feedback and set small vegetable goal<br>3. Main topic is low fat dairy: a) brief assessment on how much you are eating, b) confidence in limiting, c) barrier assessment (lack of time, similar	1. Review top 4 most important goals that participant selected at baseline, participant selects a different life goal to discuss<br>2. Discuss how that goal affects a goal of eating healthy (easier, harder, neither)<br>3. Provide diet tips to help with

	to above) d) Planning ahead to overcome barriers<br>4. Goal setting: low-fat dairy<br>5. Closing: take away and reminder about vegetable goal	highest ranking resource management issue (time, money, physical energy, mental energy).
Mo. 4<br>Call 8	1. Follow-up on dairy goal<br>2. Assess: assessment of fruit and feedback – and ask to add fruit for a small goal<br>3. Main topic is oils, spreads, added fat: a) brief assessment on how much you are eating, b) confidence in limiting, c) barrier assessment (lack of time, Don't know how to prepare lower fat foods; Someone else prepares my food; Don't like the taste of low-fat foods; Craving for fats and oils is strong; Habit; in the house; Eat out a lot) d) Planning ahead to overcome barriers<br>4. Goal setting: Reducing oils/using healthy oils<br>5. Closing: take away and reminder about fruit goal	1. Monitoring of Nutrition Goals similar to TLC-EAT with tips for improving.<br>2. Goal management topics discussed. Participant chooses to discuss either goal conflict, shielding, facilitation, or redundancy.
Mo. 5<br>Call 9	1. Follow-up on oils and/or fruit goals<br>2. Assess: eating out and spreads; feedback and suggestions<br>3. Main topic is beverages (sugary drinks) : a) assess how much, b) confidence in limiting, c) barrier assessment (lack of time, similar to previous) d) Planning ahead to overcome barriers<br>4. Goal setting: limiting sugary drinks<br>5. Closing: take away on topics covered	1. Review top 4 most important goals that participant selected at baseline, participant selects a different life goal to discuss<br>2. Discuss how that goal affects a goal of eating healthy (easier, harder, neither)<br>3. Provide diet tips to help with highest ranking resource management issue (time, money, physical energy, mental energy).
Mo. 6<br>Call 10	1. Fun quiz about recommendations and healthy eating<br>2. Follow-up on goal set last call<br>3. Recap of the last 6 months: Positive feedback on how far the participant has come and reminder of healthy eating recommendations<br>4. Reminder that it is the last call and	1. Participant reminded about each of the GST principles and asked to choose one to hear about (e.g., goal conflict, shielding, facilitation, redundancy).<br>2. Vignette with Jack or Jill

good bye

Table 3. Participants rank ordered 15 life goals during assessments; the top four ranked goals were sent to TLC-GST for tailored feedback and discussion on calls

1. Take care of a physical or mental health condition
2. Improve or keep up the appearance of my home/apartment
3. Taking Care of Extended Family (parents, grandparents, relatives, friends)
4. Be a better parent or grandparent
5. Spend quality time with friends and/or loved ones,
6. Be active in my church or place of worship
7. Work on improving my community or Help others in need
8. Make time for the things I really enjoy doing such as hobbies, music, reading, art, dancing
9. Get more education or another degree
10. Learn new things perhaps by taking a course, attending a workshop, or reading a book
11. Exercise Regularly
12. Be well dressed, have a pleasing appearance, or to look good
13. Doing Well at Work
14. Earn more money / Get a new or better job
15. Saving Money or investing for the future
